# A scoping review regarding reproductive capacity modulation based on alpha-ketoglutarate supplementation

**DOI:** 10.1530/REP-24-0137

**Published:** 2024-10-07

**Authors:** Bogdan Doroftei, Ovidiu-Dumitru Ilie, Sergiu Timofeiov, Ana-Maria Dabuleanu, Ioana-Sadyie Scripcariu, Romeo Micu, Elena Tataranu

**Affiliations:** 1 Department of Mother and Child, Faculty of Medicine, University of Medicine and Pharmacy Grigore T. Popa, Iasi, Romania; 2 Clinical Hospital of Obstetrics and Gynecology Cuza Voda, Iasi, Romania; 3 Origyn Fertility Center, Iasi, Romania; 4 Department of Surgery, Faculty of Medicine, University of Medicine and Pharmacy Grigore T. Popa, Iasi, Romania; 5 3rd Surgical Unit, St. Spiridon County Emergency Clinical Hospital, Iasi, Romania; 6 Department of Human Assisted Reproduction of 1st Gynecology Clinic, University of Medicine and Pharmacy Iuliu Hatieganu, Cluj-Napoca, Romania; 7 Faculty of Medicine and Biological Sciences, Stefan cel Mare University of Suceava, Suceava, Romania

## Abstract

**In brief:**

Alpha-ketoglutarate is a common metabolite in the tricarboxylic acid cycle and is central in modulating the reproductive potential in animal models. The present scoping review systematically covers the spectrum of a wide range of evidence from different viewpoints, focusing on the underlying processes and mechanisms of the developmental framework, aiming to fill the gaps within the existing literature.

**Abstract:**

Alpha-ketoglutarate is an important intermediate molecule in the tricarboxylic acid cycle with a prominent role in distinct biological processes such as cellular energy metabolism, epigenetic regulation, and signaling pathways. We conducted a registered scoping review (OSF: osf.io/b8nyt) to explore the impact of exogenous supplementation on reproductive capabilities. Our strategy included evaluating the main research literature from different databases like PubMed-MEDLINE, Web of Science^TM^, Scopus, and Excerpta Medica dataBASE using a specific systematic layout to encompass all investigations based on experimental models and critically compare the results. Twenty-one studies were included in the main body of this manuscript, which revealed that exogenous supplementation induced dose- and sex-dependent modifications. This metabolite modulates the expression of pluripotency genes, thus controlling stem cells’ self-renewal, differentiation, and reprogramming dynamics, while also alleviating structural transformations induced by exposure to heavy metals and other inhibitors. This significantly demonstrated a direct influence of alpha-ketoglutarate in mitigating oxidative stress and prolonging the lifespan, consequently supporting metabolic and endocrine adjustments. It influences oocyte quality and quantity, delays reproductive aging, and establishes an optimal competence framework for development with minimal risk of failure. Therefore, alpha-ketoglutarate is linked to improving reproductive performance, but further studies are needed due to a lack of studies on humans.

## Introduction

Alpha-ketoglutarate (α-KG) is an essential intermediate metabolite in the tricarboxylic acid (TCA) cycle, also regarded as the Krebs or citric acid cycle (Harrison & Pierzynowski 2008, Wu *et al.* 2016). This endogenous α-ketoglutaric acid anion actively participates as a factor in fundamental biological processes linked to cellular energy metabolism, epigenetic regulation, and signaling pathways (Zdzisińska *et al.* 2017, Legendre *et al.* 2020). α-KG may be formed and dissociated through several pathways: (I) isocitrate oxidative decarboxylation, catalyzed by isocitrate dehydrogenase (IDH), (II) oxidative deamination of glutamate (Glu) via glutamate dehydrogenase (GLDH) (III) or decarboxylation to succinyl-coenzyme A (CoA) and carbon dioxide (CO_2_) by α-KG dehydrogenase (Wu *et al.* 2016, Zdzisińska *et al.* 2017, Liu *et al.* 2018*a*, Legendre *et al.* 2020).

Studies have demonstrated that physiological impairments are directly correlated with age, marked by a decline in the quality and quantity of oocytes (te Velde & Pearson 2002) complementary to the excess of free radicals assembling (Guimarães *et al.* 2021). Subsidiary investigations recommended incorporating α-KG into the dietary routine (Velvizhi *et al.* 2002, Jiang *et al.* 2017) to retard aging (Demidenko *et al.* 2021) as a viable alternative to prolong life’s longevity and health (Asadi Shahmirzadi *et al.* 2020).

The intricate interplay between energy metabolism and cell signaling pathways has been thoroughly substantiated (Wu *et al.* 2016, Liu *et al.* 2018*a*, Legendre *et al.* 2020). Therefore, exogenous supplementation might strengthen the antioxidant defenses against reactive oxygen species (ROS) via decarboxylation (Mailloux *et al.* 2009, Bignucolo *et al.* 2013, Aldarini *et al.* 2017). Besides its scavenger effect (Song *et al.* 2016, Wu *et al.* 2019) to avert premature aging (Goud *et al.* 2008, Lord & Aitken 2013, Sasaki *et al.* 2019), α-KG maintains the integrity of DNA, regulates autophagy and apoptosis (Satpute *et al.* 2010, Gibson *et al.* 2012, An *et al.* 2021, Qin *et al.* 2021), and alleviates inflammatory reactions and abnormal immune responses (Liu *et al.* 2018*b*). Available data highlighted the reliability of α-KG for reproductive disturbances based on the numerous applications that targeted ROS’ elevated ratio consequences on a molecular and morphological level in affected oocytes and on altered mitochondria (Zhang *et al.* 2019*a*, Yang *et al.* 2020). Conversely, the current state of knowledge enabled applications on abnormal morphology and early embryonic development decline (Zhang *et al.* 2019*a*,*b*, Miao *et al.* 2020) or correlated with depletion (Balaban & Urman 2006, Miao *et al.* 2009).

These structural transformations might have multiple negative outcomes that range from a heightened risk of aneuploidy (Mikwar *et al.* 2020) or miscarriage (Santonocito *et al.* 2013, Capalbo *et al.* 2017), to reduced testosterone secretion, spermatogenesis, and sperm motility and morphology abnormalities (Harris *et al.* 2011). Despite controversies over the passive influence of metabolism in embryogenesis, cell fate, and signal transduction (Wu *et al.* 2016, Zdzisińska *et al.* 2017, Legendre *et al.* 2020), recent discoveries promoted advances dedicated to containing oocyte rate decrease (Lord & Aitken 2013, Zhang *et al.* 2019*a*,*b*) caused by telomere shortening (Broekmans *et al.* 2009).

Therefore, this scoping review aims to provide an updated perspective on the latest applications to reconfigure reproductive capacity. The need for this manuscript derives from the limited settings compared to other research directions (Meng *et al.* 2022, Naeini *et al.* 2023). Conclusively, the main objective is to open possible new therapeutic approaches and translate gained information into clinical practice.

## Materials and methods

### Methodology and registration

The protocol of this manuscript was designed to adhere to the Preferred Reporting Items for Systematic Reviews and Meta-Analyses 2018 guidelines for Scoping Review (PRISMA-ScR) (Munn *et al.* 2018, Tricco *et al.* 2018) and was registered in the Open Science Framework (OSF) with the ID: osf.io/b8nyt (https://doi.org/10.17605/OSF.IO/YT3BA).

### Ethics committee

This manuscript did not require Institutional Review Board (IRB) approval, signed consent forms, or a third-party evaluation because data was extracted from published studies.

### Objectives

We hypothesized that adequate supplementation with α-KG may improve the ability of the reproductive system, similar to its involvement in extending the lifespan and reversing oxidative stress (OS) following the normalization of ROS.

### Research questions

#### Primary question

Does the supplementation with α-KG enhance reproduction in experimental laboratory models?

*Secondary questions*

Does the supplementation with α-KG prevent OS by normalizing the ROS levels?

Does the supplementation with α-KG extend the lifespan?

### Source databases

We conducted searches to encompass the latest critical literature from January 1, 2010, to March 1, 2024, by accessing primary electronic academic databases: PubMed-MEDLINE—United States National Library of Medicine (NLM, 1996), Web of Science^TM^ (WOS) (Clarivate Analytics, 1997), Scopus (Elsevier, 2004) (Falagas *et al.* 2008), and Excerpta Medica dataBASE (EMBASE) (Elsevier, 1947) (accessed on March 8, 2024).

### Search strings

We applied dedicated and controlled scientific terminology using MeSH (Medical Subject Headings), Boolean operators (‘AND’ or ‘OR’ or ‘NOT’), and Emtree terms. This approach was meant to ensure comprehensive coverage of relevant research articles before undergoing the identification, collection, ranking, and analysis steps. Several main input terms such as ‘α-ketoglutaric acid,’ ‘alpha-ketoglutaric acid,’ ‘2-ketoglutaric acid,’ ‘2-oxoglutaric acid,’ ‘oxoglutaric acid,’ and ‘2-oxopentanedioic acid,’ excepting ‘AKG’ and ‘2-oxoglutamate,’ were appointed as (Major Topic) with the MeSH Unique ID: D007656, Tree Number(s): D02.241.081.337.351.502, D02.241.755.465 and applied for distinct fields. The primary subtopics included ‘reproductive system’ (‘Genitalia’ (MeSH) – ID: D005835, Tree Number(s): A05.360), ‘fertility’ (‘Fertility’ (MeSH) – ID: D005298, Tree Number(s): G08.686.210), and ‘reproduction’ (‘Reproduction’ (MeSH) – ID: D012098, Tree Number(s): G08.686.784) in parallel with ‘laboratory model’ (‘Models, Animal’ (MeSH) – ID: D023421, Tree Number(s): E05.598) and ‘experimental animal’ (‘Animals, Laboratory’ (MeSH) – ID: D000830, Tree Number(s): B01.050.050.199) as found on the NLM official website (accessed on March 8, 2024). The complete strings for each database can be found in Supplementary File 1 (see section on supplementary materials given at the end of this article).

### Study selection

The references list was subsequently imported to Mendeley – Reference Management Software (v. 1.19.8) (Elsevier, 2013) and de-duplicated using the ‘check for duplicates’ function followed by additional manual screening. Each investigator independently reviewed the titles ± abstracts of individual retrieved records. Afterward, BD, O-DI, A-MD, I-SS, and RM assessed the abstracts and the full-length content of each qualifying article. Possible conflicting opinions were settled unanimously by common consent with BD, ST, and ET.

### Extraction of data

General data from the retrieved studies were organized in a tabular standardized form using Microsoft Excel 2010 (Microsoft Corporation, Redmond, WA, USA) by BD, O-DI, A-MD, I-SS, and RM that included methodological characteristics such as experimental model/cell line, compound, concentration, supplementation method, and main observations.

### Criteria of inclusion and exclusion

Research had to be written exclusively in English and contain original data obtained from experiments carried out on experimental laboratory models. A particular eligibility requirement was that the studies had to include models with genetic similarity comparable to humans for clinical relevance or cell cultures to deepen the understanding of the underlying mechanisms further. Any other type of manuscript was automatically removed.

## Results

### Number of results and final inclusion

**Figure 1:**
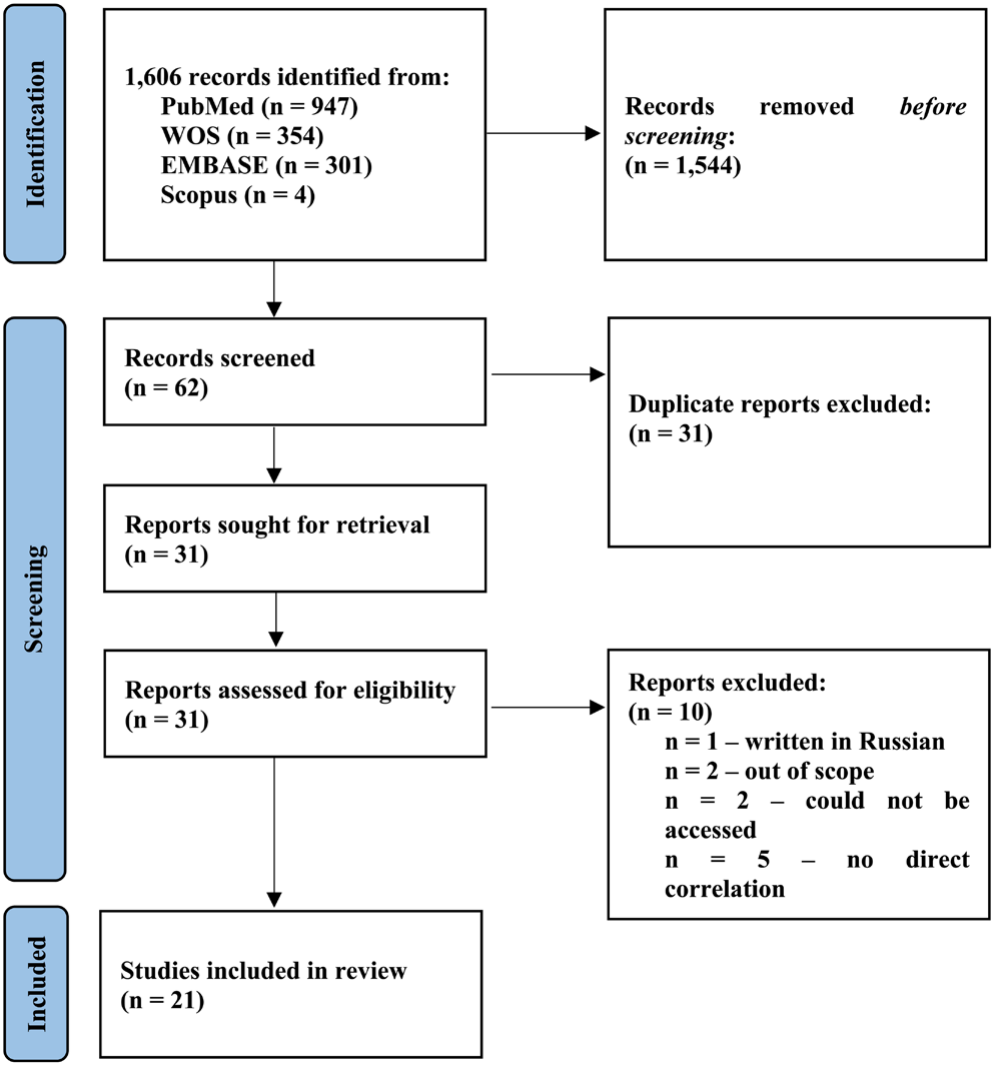
PRISMA-ScR diagram.

A total of 1606 studies were returned in the pre-determined timeframe, of which 947 (58.96%) were from PubMed-MEDLINE, 354 (22.04%) from WOS^TM^, 301 (18.74%) from EMBASE, and four (0.24%) from Scopus (Supplementary File 2) (accessed on March 8, 2024). By removing 1544 studies that did not undergo review, 62 studies remained, of which 31 preliminary eligible papers were retained, excluding duplicates. Furthermore, ten articles were removed in the second step due to the following reasons: one was an article written in Russian (Lucenko *et al.* 2016), two were out of scope (Willenborg *et al.* 2009, Mariño *et al.* 2014), two were eligible but the full content could not be accessed (Zhang *et al.* 2019*a*, Penn *et al.* 2023) and five had no direct correlation (Pedroso *et al.* 2019, Dobrowolski *et al.* 2021, Sekita *et al.* 2021, Tanaka *et al.* 2021, Shibata *et al.* 2024) (Fig. 1). Therefore, in the 21 studies that met the eligibility criteria, one was conducted on ovine (Hao *et al.* 2022), two on porcine (Breininger *et al.* 2014, Chen *et al.* 2022), four on *Drosophila melanogaster* (Bayliak *et al.* 2017, 2019, Lylyk *et al.* 2018, Su *et al.* 2019), and 14 on murine models: two on mice (Zhang *et al.* 2021, Wang *et al.* 2023), three on rats (Li *et al.* 2010, 2023, Liu *et al.* 2024) and nine on stem cells that investigated the pluripotency, differentiation, or reprogramming modifications in standard or transgenic lines (Carey *et al.* 2015, Hwang *et al.* 2016, TeSlaa *et al.* 2016, Choi *et al.* 2019, Tischler *et al.* 2019, Xing *et al.* 2020, Xu *et al.* 2022, Yang *et al.* 2022, Tang *et al.* 2023) (Supplementary Table 1).

### Study features

Half of the studies were conducted exclusively by researchers from China (50%) (Li *et al.* 2010, 2023, Su *et al.* 2019, Xing *et al.* 2020, Chen *et al.* 2022, Hao *et al.* 2022, Yang *et al.* 2022, Xu *et al.* 2022, Tang *et al.* 2023, Wang *et al.* 2023, Liu *et al.* 2024), Japan (9.09%) (Choi *et al.* 2019, Tanaka *et al.* 2021), USA (9.09%) (Carey *et al.* 2015, TeSlaa *et al.* 2016) Argentina (4.54%) (Breininger *et al.* 2014), Republic of Korea (4.54%) (Hwang *et al.* 2016) or multidisciplinary teams from Ukraine and Canada (13.63%) (Bayliak *et al.* 2017, 2019, Lylyk *et al.* 2018), United Kingdom and Germany (4.54%) (Tischler *et al.* 2019) or with double affiliations from China and United Kingdom (4.54%) (Zhang *et al.* 2021). The reiterative lists of each assessment phase can be found in Supplementary File 3.

### Stem cell pluripotency and differentiation

Although the connection between cellular metabolism and cell differentiation has been recently addressed, auxiliary research uncovered the potential of self-renewal and differentiation (Carey *et al.* 2015, Hwang *et al.* 2016, TeSlaa *et al.* 2016, Tischler *et al.* 2019, Xing *et al.* 2020, Yang *et al.* 2022, Tang *et al.* 2023). Naïve mESCs preserve their pluripotency until reaching the PGCs state (Tischler *et al.* 2019) even in the absence of Gln (Carey *et al.* 2015). In this context, supplementation with α-KG concomitantly with glucose or pyruvate sustains the Gln-Glu-α-KG axis to support maternal decidualization (Tang *et al.* 2023), blastocyst formation (Choi *et al.* 2019), and the amount of intracellular α-KG (Carey *et al.* 2015) through the IDH2-mediated production (Tischler *et al.* 2019). α-KG delays aging and heat shock-induced changes upon sperm via OXGR1 expressed in epididymal SMCs (Xu *et al.* 2022), and restores Gln-Glu flux metabolism (Tang *et al.* 2023). Several remarks underlined the inhibition of the BPTES effect on PGCLC (Xing *et al.* 2020) which translates into the number and function of dNK (Yang *et al.* 2022) that ultimately may reduce the risk of induced pregnancy (Yang *et al.* 2022) and spontaneous miscarriage (Tang *et al.* 2023). Naïve mESCs store α-KG/succinate (Carey *et al.* 2015), which is regulated by Psat1 (Hwang *et al.* 2016) and required for primed PSCs acceleration (TeSlaa *et al.* 2016) and epigenetic reprogramming (Carey *et al.* 2015, Hwang *et al.* 2016, TeSlaa *et al.* 2016). Psat1 KD is thought to be the cause of reduced mESCs DNA 5’-hydroxymethylcytosine (5’-hmC) methylation (Hwang *et al.* 2016), influencing the ATP generation mechanism (TeSlaa *et al.* 2016, Tang *et al.* 2023) and H3K27me3-based chromatin modifications and ten-eleven translocation (TET)-dependent DNA demethylation process (Carey *et al.* 2015, Hwang *et al.* 2016, TeSlaa *et al.* 2016, Tischler *et al.* 2019, Xing *et al.* 2020, Yang *et al.* 2022, Tang *et al.* 2023).

### Rodents

While higher mammals are subjected to reproductive aging mainly because of telomere shortening (Zhang *et al.* 2021), controlled concentrations of α-KG might delay the decline in ovarian reserve (Li *et al.* 2023, Wang *et al.* 2023), and loss of function (Zhang *et al.* 2021, Liu *et al.* 2024). This metabolite triggers adaptations of metabolism and consequent variations in levels of hormones (Li *et al.* 2023, Liu *et al.* 2024), linked to reproductive function reconfiguration (Zhang *et al.* 2021) even in aging oocytes post-ovulation (Wang *et al.* 2023). α-KG inhibits the mTOR pathway by blocking ATP synthase (Zhang *et al.* 2021), thus contradicting previous studies showing higher ATP levels (Li *et al.* 2023) due to enhanced mitochondrial membrane potential (Wang *et al.* 2023). Although the level of pyruvate in females is reduced (Li *et al.* 2023) unlike males who experience an increase in O_2_^-.^ level in sperm, this does not always have negative effects (Liu *et al.* 2024) since both are pivotal in sustaining spermatozoa ATP levels for motility (Li *et al.* 2010). These antioxidants are involved in diminishing ROS levels, which thus mitigates the pro-inflammatory cascade (Liu *et al.* 2024), resulting in a lower rate of fragmentation, abnormal spindle assembly (Wang *et al.* 2023), and apoptosis (Li *et al.* 2023).

### Flies

*Drosophila melanogaster* is a genetic model broadly employed for deepening our awareness of the reproduction paradigm (Bayliak *et al.* 2017, Lylyk *et al.* 2018, Su *et al.* 2019) and the consequences of heavy metals exposure (Bayliak *et al.* 2019). α-KG diet supplementation leads to fluctuations in metabolic parameters dependent on the developmental stage (Bayliak *et al.* 2017) and effectively alleviates AlCl_3_ toxicity (Bayliak *et al.* 2019). The extended lifespan seen at specific concentrations is dose- and sex-dependent (Lylyk *et al.* 2018, Bayliak *et al.* 2019, Su *et al.* 2019), but higher doses can be harmful, especially in males (Lylyk *et al.* 2018). This does not exacerbate the OS but triggers adaptive responses instead (Bayliak *et al.* 2017, 2019, Lylyk *et al.* 2018, Su *et al.* 2019) due to decreased ATP/ADP ratio (Su *et al.* 2019). This might clarify the heightened tolerance to heat stress (Bayliak *et al.* 2017, 2019), upregulation of the mRNA expression of protein genes involved in longevity (Su *et al.* 2019), and decreased fecundity (Bayliak *et al.* 2017, 2019, Lylyk *et al.* 2018, Su *et al.* 2019), while remarkably enhancing the egg-laying capacity in AlCl_3_-reared flies (Bayliak *et al.* 2019).

### Porcine

Research indicates that α-KG is a potent scavenger of free radicals in porcine, as it significantly reduces ROS generation required for the biosynthesis of GSH. The NRF2/ARE pathway facilitates the transcription of apoptotic genes and enhances mitochondrial function by activating NRF2, thus preventing a biased process of apoptosis (Chen *et al.* 2022). α-KG promotes an optimal framework for pig embryo development through the upregulation of pluripotency genes, leading to the formation of blastocysts and the total cell number during in vitro maturation (IVM) (Chen *et al.* 2022). Nonetheless, the addition in the culture medium of phosphofructokinase (PFK) and malate dehydrogenase (MDH) can hinder the nutritional requirements of cumulus-oocyte complex (COC) during IVM and cause dysfunction of meiotic maturation (Breininger *et al.* 2014).

### Ovine

In contrast to results from the fruit flies, dm-α-KG was found to induce comparable phenotypical features as seen in porcine, notably an increased ATP synthesis based on enhanced mitochondrial activity and GSH production against pro-oxidants. dm-α-KG relieves abnormal ROS generation, which could potentially degrade the integrity of the DNA, alter mitochondria, and trigger apoptosis. Therefore, dm-α-KG showed promising activity in maintaining nuclear maturation rate, CGs dynamic, and embryonic developmental competence (Hao *et al.* 2022).

## Discussion

**Figure 2:**
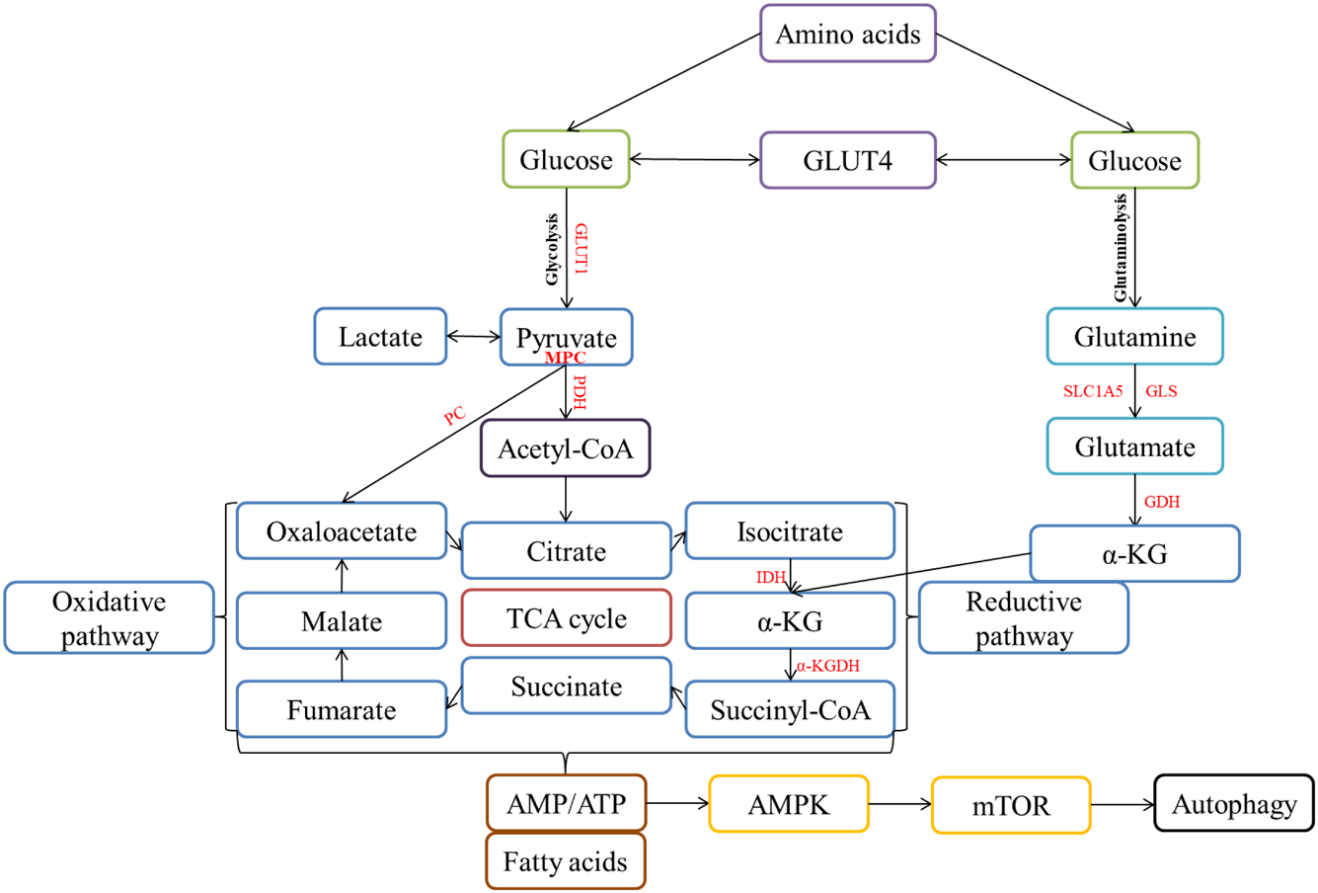
Schematic overview of mitochondrial metabolism of pyruvate and glutamine in the TCA cycle. Amino acids-derived glucose is subjected to sequential phases of conversion that facilitate the production of α-KG through either glycolysis or glutaminolysis. The final fatty acids are produced by blocking the oxidative pathway and enhancing the reductive pathway. The AMPK and mTOR signaling pathways control the α-KG level when the ratio of AMP/ATP is high, thus regulating autophagy. GLUT1/4 – glucose transporter type 1/4, MPC – mitochondrial pyruvate carrier, PDH – pyruvate dehydrogenase, PC – pyruvate carboxylase, IDH – isocitrate dehydrogenase, α-KGDH – α-KG dehydrogenase, GLS – glutaminase, GDH – glutamate dehydrogenase.

α-KG is a source of ATP in all living organisms’ cells (He *et al.* 2015) and is predominantly found in mitochondria and cytosol (Fig. 2) besides the bloodstream (Wagner *et al.* 2010). Previous experiments on animal models demonstrated high versatility (Niemiec *et al.* 2011, Radzki *et al.* 2012, 2015, Wang *et al.* 2015, Cai *et al.* 2018) in various organs such as the brain, heart, liver, gastrointestinal tract, skeletal muscle, and adipose tissue (He *et al.* 2015). It has been proven to modulate the molecular landscape via the mechanistic target of rapamycin (mTOR) and AMP-activated protein kinase (AMPK) (He *et al.* 2015), and even hypothesized to interact with calcium/calmodulin-dependent protein kinase kinase 2 (CamKK2) (Jin *et al.* 2018).

mTOR is part of a conserved group of serine/threonine (Ser/Thr) protein kinases of the phosphatidylinositol 3-kinase-related kinase (PIKK) family, comprising rapamycin-sensitive complex 1 (mTORC1) and relatively rapamycin-insensitive complex 2 (mTORC2). Both play a role in growth and metabolism, primarily found in the nucleus and cytoplasm (Saxton & Sabatini 2017, Jhanwar-Uniyal *et al.* 2019), while AMPK serves as a key energy sensor in aging and lifespan (Hardie *et al.* 2012, Huang *et al.* 2013), collectively promoting lysosomal translocation through mTORC1 known to block autophagy (Durán *et al.* 2012).

The initial data on *Caenorhabditis elegans’* increased lifespan from inhibiting ATP synthase and phosphor-Akt (Shrimali *et al.* 2021), as well as the mTOR signaling pathway (Hou *et al.* 2010, Żurek *et al.* 2019) by α-KG in a dose-dependent manner, was founded a decade ago (Chin *et al.* 2014). Noteworthy, α-KG longevity partially depends on AMPK and the forkhead box O (FOXO) (Urban *et al.* 2007) as already seen in flies (Kapahi *et al.* 2004, Luong *et al.* 2006, Su *et al.* 2019) and mice (Selman *et al.* 2009).

It is important to note that AMPK activates when the AMP/ATP ratio is high and hampers TOR signaling by phosphorylation of the TOR suppressor tuberous sclerosis complex (TSC) 1, which regulates cell energy rate and metabolism (Toivonen *et al.* 2007). α-KG activate mTOR in the intestinal epithelial cells of pigs to stimulate protein (Yao *et al.* 2012) or milk protein synthesis (Jiang *et al.* 2016).

α-KG can increase the FOXO mRNA expression (Su *et al.* 2019), a transcription factor that helps maintain the cytosolic level of α-KG (Charitou *et al.* 2015) by regulating isocitrate dehydrogenase 1 (IDH1). Prolyl hydroxylase domain protein (PHD) catalyzes proline hydroxylation and facilitates FOXO3 degradation. A possible reduction of α-KG in hypoxic tubules instead stabilizes FOXO3 to augment autophagy (Tran *et al.* 2019).

Biological aging involves a high degree of epigenetic changes (Shi & Whetstine 2007, Walport *et al.* 2012, Salminen *et al.* 2015, Martínez-Reyes & Chandel 2020), that implicitly lead to hypermethylation of specific DNA regions and histone patterns (Gonzalo, 2010, Sierra *et al.* 2015). Despite the recent progress in translational medicine (Xiao *et al.* 2016, Gyanwali *et al.* 2022, Naeini *et al.* 2023), the clinical validation of α-KG as a feed additive is still lacking as existing data only includes experiments conducted in animal models. Nonetheless, direct oral supplementation may be a feasible method to address a deficiency as demonstrated by (Demidenko *et al.* 2021) who successfully reduced the average biological aging by eight years in both sexes following Rejuvant^®^ tablets for seven months (*P* = 6.538x10^-12^). 2-oxoglutarate, regarded as α-KG, along with other Krebs cycle analogs succinate, fumarate, and 2-hydroxyglutarate (2-HG) are integral to mitochondrial metabolism and influence DNA and histone methylation (Ward & Thompson 2012, Badeaux & Shi 2013, Kaelin & McKnight 2013, Salminen *et al.* 2014*a*,*b*). These essential epigenetic regulators of gene expression are part of the 2-oxoglutarate-dependent dioxygenases (2-OGDDs) family, known for their involvement in demethylation and hydroxylation processes (McDonough *et al.* 2010, Loenarz & Schofield 2011) which might shape chromatin structure and function (Ward & Thompson 2012, Badeaux & Shi 2013, Kaelin & McKnight 2013).

Precisely, hydroxylases TET1-3 are DNA demethylases that catalyze the oxidative decarboxylation of α-KG to convert 5-methylcytosine (5-mC) into 5-hydroxymethylcytosine (5-hmC) and trigger demethylation of CpG loci in DNA (Tahiliani *et al.* 2009, Ito *et al.* 2010, Salminen *et al.* 2015). Similarly, histone demethylases containing Jumonji C domain lysine KDM2-7 contribute to the regulation of chromatin landscape changes associated with aging (Shi & Whetstine 2007, Walport *et al.* 2012, Salminen *et al.* 2015). As a result, they are accountable for removing methyl groups from specific methylation sites in histones, a process that also catalyzes the demethylation of trimethylated lysines and arginines (Hoffmann *et al.* 2012), crucial for forming both transcriptionally active and inactive chromatin (Shi & Whetstine 2007, Gonzalo 2010, Walport *et al.* 2012).

Data from cancer research showed that succinate and fumarate could act as competitive inhibitors of the 2-OGDO enzymes, precisely KDMs and TETs, which potentiate methylation of DNA and histones by methyltransferases, ultimately causing carcinogenesis (Ward & Thompson 2012, Kaelin & McKnight 2013, Martínez-Reyes & Chandel 2020). Considering the extensive work of (Salminen *et al.* 2014*a*,*b*, 2015) on the topics discussed earlier, the role of KDM2/6 in gene expression and cell fate is rather complex as it involves both inducing (Agherbi *et al.* 2009, Perrigue *et al.* 2015) and inhibiting senescence (Pfau *et al.* 2008) through the cell cycle arrest mediators p53 and pRb (Leon & Aird 2019).

In conclusion, chronic inflammation could be caused by enhanced cellular senescence leading to premature aging (Agherbi *et al.* 2009, Perrigue *et al.* 2015, Salminen *et al.* 2015), while histone demethylases containing Jumonji C domain lysine up-regulation are linked to cancer progression by blocking senescence (Pfau *et al.* 2008, Wang *et al.* 2018, Leon & Aird 2019).

Understanding the reproduction phenomenon to counter the decrease in quality and quantity of oocytes (te Velde & Pearson 2002) is mandatory, especially in conjunction with early embryonic potential (Zhang *et al.* 2019*a*,*b*, Miao *et al.* 2020), to reduce the risk of aneuploidy (Mikwar *et al.* 2020) and miscarriage (Santonocito *et al.* 2013, Capalbo *et al.* 2017) in light of the numerous applications targeting aged oocytes (Miao *et al.* 2009, Yamada-Fukunaga *et al.* 2013, Garcia *et al.* 2019). The telomerase complex, which consists of Terc and the catalytic subunit Tert, plays a compensatory role in slowing aging by supporting the maintenance of the telomere system along with SIRT6 (Tennen *et al.* 2011), as well as performing important roles in DNA repair and inflammation (You & Liang 2023).

In the urge for an effective antioxidant against ROS, which is seen as the main forerunner of decreased quality of aged oocytes (Lord & Aitken 2013, Sasaki *et al.* 2019), α-KG has emerged as the key factor in refining the antioxidant defense system (Velvizhi *et al.* 2002) that is also responsible for removing H_2_O_2_ by decarboxylation (Aldarini *et al.* 2017). However, α-KG via the Nrf2 pathway in adequate concentrations counteracts the impact of brutasol and partially restores metal content balance (Orgad *et al.* 1998, Wu *et al.* 2012, Fu *et al.* 2014, Maya *et al.* 2016) after a 26% increase of aconitase (Middaugh *et al.* 2005, Wu *et al.* 2012) to support embryonic development and shield oocytes from OS (Jiang *et al.* 2021). An elevated intracellular level of GSH (Brigelius-Flohé, 1999, de Matos & Furnus 2000, Liu *et al.* 2018*a*) prevents DNA damage and cell death (Jia *et al.* 2018, Zhou *et al.* 2019, Chen *et al.* 2020, Aghaz *et al.* 2021, El-Sheikh *et al.* 2021, Min *et al.* 2021), indicating an ideal framework for developmental competence (Ren *et al.* 2021). Alternatively, α-KG may influence the PFK and MDH-related COC role on oocyte maturation in bovine (Cetica *et al.* 2002, 2003), and porcine (Breininger *et al.* 2014) as previously suggested, owing that a preliminary investigation that revealed the presence of IDH-nicotinamide adenine dinucleotide (NAD) in porcine COC but not in cows (Cetica *et al.* 2003).

According to the available information regarding α-KG’s potential to sustain both naïve and primed PSCs’ pluripotency and differentiation (Leitch *et al.* 2013), a biphasic concept of α-KG was proposed, modulated by the oxygen level apart from its position as a cofactor for DNA and histone demethylation (TeSlaa *et al.* 2016). The TET complex is a widely recognized mechanism in DNA demethylation known to participate in converting 5-methylcytosine (5-mC) to 5-hydroxymethylcytosine (5-hmC), 5-formylcytosine (5-fC), and 5-carboxylcytosine (5-caC) (TeSlaa *et al.* 2016). Maternal high-fat diet (MHFD) reduces 5-hmC DNA methylation while increasing 5-fC (Penn *et al.* 2023), hindering early-stage embryo development that depends on low ATP production and altered activity of DNA methyltransferase 3 alpha (Dnmt3a) and an elevated 5-hmC and 5-mC ratio (Zhang *et al.* 2019*a*). The ratio is notably elevated in PSCs compared to differentiated cells (Tran *et al.* 2019), thus confirming epigenetic reprogramming based on the TET-mediated conversion of 5-mC to 5-hmC (Sekita *et al.* 2021).

Studies have shown that Jumonji C-containing domains that reunite lysine demethylase 3-6A/3-6B (KDM3A/3B/6A/6B) (Ko *et al.* 2006, Loh *et al.* 2007) (JHDMs) require α-KG to target distinct histones (Klose & Zhang 2007). TET members methylcytosine dioxygenase 1 (TET1) and TET methylcytosine dioxygenase 2 (TET2) (Costa *et al.* 2013, Hackett & Surani 2014) can maintain the naïve pluripotency epigenetic state by demethylating H3K27me2/me3 (histone 3 lysine 27 methylation) (TeSlaa *et al.* 2016) given that low DNA demethylation levels may result in an increased spread of H3K27me3 at high CpG regions (Zylicz *et al.* 2015). α-KG could potentially preserve the naïve pluripotent status in PSCs when 2i inhibitors are substituted (Ying *et al.* 2008, Tischler *et al.* 2019) due to IDH-related high expression based on DNA methyltransferase 3 beta (DNMT3B) depletion. Some suggest that a re-wiring of 2i in culture conditions could support Gln-independent growth of naïve mESCs (Carey *et al.* 2015).

The switch from naïve mESCs to PGC-competent EpiLCs involves a change to a glycolytic state instead of oxidative (Zhou *et al.* 2012, Zhang *et al.* 2016) during later stages (Folmes *et al.* 2012) due to the upregulation of lin-28 homolog B (Lin28b). Lin28b is a protein-coding gene involved in oxidative suppression and regulation of glucose metabolism (Zhu *et al.* 2011, Zhang *et al.* 2016) as the metabolic modulator 2-DG is activated (Hayashi *et al.* 2017). Lactate shuttle (LS) and Warburg-like glycolysis participate during decidualization since DNA methylation is stable (Maekawa *et al.* 2019, Liu *et al.* 2020) and DNA hydroxylases TET are dioxygenases (Raffel *et al.* 2017) dependent on α-KG. As the bio-energetic requirements (Zuo *et al.* 2015, Huang *et al.* 2019, Tamura *et al.* 2021) are assured, supplementary resources for proliferation and differentiation are necessary despite higher glucose intake (Ying *et al.* 2021), these conditions under an aerobic state being met by Gln metabolism (Le *et al.* 2012, Li & Zhang 2016).

α-KG can also trigger primed PSCs differentiation by interacting with chaperone-mediated autophagy (CMA) (Xu *et al.* 2020), causing changes in the expression of transcription factors and being limited by the inhibitory effects of 3-nitropropionic acid (NPA). This reduction in succinate delays PSCs differentiation, but this effect is counteracted by TET2, which promotes differentiation (Hon *et al.* 2014, TeSlaa *et al.* 2016). Autophagy collaborates with the ubiquitin-proteasome system (UPS) to regulate protein levels linked to pluripotency and influences the self-renewal or differentiation of ESCs based on CMA level (Xu *et al.* 2020, Xu & Yang 2022).

SOX2 and OCT4 influence ESCs (Xu *et al.* 2020) through inhibition of lysosome-associated membrane protein type 2A (LAMP2A), a key component in parallel with cytosolic chaperone HSC70 that defines CMA activity (Cuervo & Dice 2000). Changes in LAMP2A levels correspond to levels of SOX2 and OCT4, and in what concerns differentiation, a possible depletion in mESCs indicates high levels of the α-KG/succinate ratio, whereas mESCs overexpression leads to a gradual decline of LAMP2A (Xu *et al.* 2020).

Finally, reprogramming has been a topic of interest on several occasions with special emphasis on TET in induced PSCs (iPSCs) from differentiated or non-pluripotent cells (Kolaczkowski & Thornton 2004, Polo *et al.* 2012, Sardina *et al.* 2018). AKT and FOXO1 signaling pathway activation or simultaneous transduction of TET2’s catalytic domain and dm-α-KG synergistically improve reprogramming (Sekita *et al.* 2021), relying on increased glycolysis and mitochondrial activity (Yu *et al.* 2014, Wilhelm *et al.* 2016).

### Strengths and limitations of the study

This primary scoping review systematically approaches this scarce topic by including multiple databases to ensure a thorough and comparative overview without neglecting critical and authoritative information. However, it should be noted that the number of applications is limited to animal studies and even lower in humans, which is why clinical trials are mandatory, setting the grounds for future randomized controlled trials (RCTs).

## Conclusions

The manuscript covers the importance of α-KG in maintaining the functionality and integrity of various biological mechanisms. Despite the research on animal models instead of humans, it is clear that α-KG supplementation improves reproductive abilities. However, additional experiments are needed to close the gap between research and clinical practice. This might result in treatment strategies and an improved understanding of the advantages of α-KG supplementation in humans.

## Supplementary Materials

Supplementary Table 1. Summarization of all applications with α-KG on reproduction performance.

Supplementary File 1. Extensive searching strategies

Supplementary File 2. Cumulative list of entries based on the year of publication and database

Supplementary File 3. Eligibility assessment

## Declaration of interest

The authors declare that there is no conflict of interest that could be perceived as prejudicing the impartiality of the research reported.

## Funding

This work did not receive any specific grant from any funding agency in the public, commercial, or not-for-profit sector.

## Data Availability Statement

The datasets used and analyzed during the current study are available from the corresponding author upon reasonable request.

## Author contribution statement

Conceptualization, Data curation, Investigation, Formal analysis, Methodology, Software, Writing – original draft: BD, O-DI, A-MD, I-SS, and RM. Supervision, Visualization, Validation, Project administration, Writing – review and editing: BD, ST, and ET. All authors have read and agreed to the published version of the manuscript.
