# Supplementary material for: A scoping review regarding reproductive capacity modulation based on alpha-ketoglutarate supplementation
Source: Reproduction. 2024 Oct 7;168(5):e240137. doi: 10.1530/REP-24-0137 (PMC11558802; doi:10.1530/REP-24-0137)
Supplement: Supplementary Table 1. Summarization of all applications with α-KG on reproduction performance. [file supplementary_table_1.pdf]

Supplementary Table 1. Summarization of all applications with α-KG on reproduction performance.

**Experimental model/ Cell line**	**Compound**	**Concentration**	**Supplementation approach**	**Main observations**	**Reference**
Sheep ovaries	dm-α-KG	0, 1.5, 3, 4.5 mM	Addition to medium	**Increased/improved in/of:** nuclear maturation rate**,** ATP synthesis**,** CGs dynamic**,** F-actin polymerization**,** mitochondrial activity**,** GSH production**,** embryonic developmental competence<br>**Decreased/reduced in/of:** ROS production**,** mitochondrial damage**,** DNA damage**,** cellular apoptosis	Hao *et al*. (2022)
Porcine oocytes	α-KG	0, 10, 20, 50, 100 µM	Addition to medium	**Increased/improved in/of:** blastocyst formation rate**,** total cell number**,** GSH production**,** mitochondrial activity<br>**Decreased/reduced in/of:** blastocyst apoptosis**,** ROS production<br>**Upregulation of:** pluripotency genes (OCT4, NANOG, SOX2)**,** anti-apoptotic gene (Bcl2) via Nrf2/ARE signaling pathway activation	Chen *et al*. (2022)
Porcine oocytes	2-oxoglutarate	0, 5, 10, 20 mM	Addition to medium	**Induced:** reduction of enzymatic activity, no effect on viability, impairment of meiotic maturation	Breininger *et al*. (2014)
Drosophila melanogaster Canton S and w^{1118}	α-KG	10 mM	Containing food	**$AlCl_3$**<br>**Increased/improved in/of:** free Fe, TAG<br>**Induced:** behavioral defects, decreased fecundity, long-term survival, metal content imbalance, decrease of glucose, mitochondrial dysfunction, OS development<br>**α-KG did not improved/ameliorated:** locomotor impairment, taste behavior defects, higher OS sensitivity, effects on glucose and TAG<br>**α-KG improved/ameliorated:** heat shock resistance, egg-laying capacity, survival, metal homeostasis, inhibition of free Fe, functional activity of aconitase, decreased intensity of OS	Bayliak *et al*. (2019)
Drosophila melanogaster w^{1118}	α-KG	1, 5, 10, 20, 100 mM	Containing food	**Larvae and young (2 days post-eclosion) females**<br>**Higher in/of:** total proteins, lipid peroxides, low molecular mass thiols, heat shock resistance, H_2O_2<br>**Lower in/of:** TAG<br>**Middle-aged females (24 days)**<br>**Higher in/of:** total proteins, glucose, TAG, heat shock resistance<br>**Lower in/of:** fecundity, OS resistance<br>**Induced:** similar OS status to the control group	Bayliak *et al*. (2017)
Drosophila melanogaster Canton S	α-KG	0, 1, 5, 10, 20 mM	Containing food	**In males**<br>**No effect in/on:** mean and maximum lifespan at 1-10 mM, maximum lifespan at 20 mM	Lylyk *et al*. (2018)

				**Induced:** increased maximum lifespan at 10 mM, shortened median lifespan at 20 mM<br>**In females**<br>**Induced:** no effect on lifespan at 1-5 mM, increased median and maximum lifespan at 10-20 mM<br>**Flies at 24 and 40 days**<br>**Increased/improved in/of:** total proteins, TAG, cold resistance<br>**Induced:** decreased fecundity, no effect on climbing activity, no resistance to OS<br>**In middle-aged (24 days) females:**<br>high levels of HSP90 protein than in the control group	
Drosophila melanogaster (w^{Dah})	α-KG	0.5, 1, 5, 10, 50, 100, 200, 1000 μM	Containing food	**α-KG did not ameliorated/prevented**<br>**Reduction in/of:** reproductive performance, ATP/ADP ratio<br>**Downregulation in/of:** *HDAC4*, *PI3K*, *TORC*, *PGC*, and *SREBP*<br>**Induced:** protection against OS, increased tolerance to starvation,<br>**α-KG improved/enhanced:**<br>**Upregulation of:** expression of heat shock genes (*Hsp22* and *Hsp70*), mRNA expression of *cry*, *FoxO*, *HNF4*, *p300*, *Sirt1* and *AMPKα*<br>**Induced:** extended lifespan, vertical climbing ability, heat shock resistance	Su *et al.* (2019)
female ICR mice	α-KG	2% (w/w)	Drinking water	**Increased/improved in/of:** ovarian reserve – follicle number, oocyte quality, mitochondrial membrane potential, post-ovulated quality of aging oocyte, early embryonic development<br>**Decreased/reduced in/of:** fragmentation rate, ROS level, abnormal spindle assembly	Wang *et al.* (2023)
female ICR mice	α-KG	2, 10, 25, 50 mM	Drinking water	**Preservation/prevention in/of:** ovarian function, quality and quantity of oocytes, telomere length<br>**Suppression/inhibition in/of:** ATP synthase, alteration of energy metabolism via mTOR pathway downregulation	Zhang *et al.* (2021)
female Sprague Dawley rats	α-KG	250 mg/kg/day	Oral gavage	**Increased/improved in/of:** BMI, ovarian index, pregnancy rate, litter size, oestradiol, lactate, ATP, RLEs of glycolysis in ovaries<br>**Decreased in/of:** serum FSH concentration, apoptosis of granulosa cells, pyruvate<br>**Induced:** partial normalization of estrous cycles, prevention of follicular loss, ovarian reserve restoration	Li *et al.* (2023)
male Sprague Dawley rats	α-ketoacids (α-KG and	4 mmol/L	Addition to medium	**H_2O_2 induced:**<br>motility impairment, depletion of ATP, sperm protein	Li *et al.* (2010)

	pyruvate), lactate, glutamate/malate			phosphorylation inhibition, acrosome reaction reduction, decreased viability<br>**Pyruvate induced:**<br>increased sperm level of $O_2^{-\cdot}$<br>**α-KG, pyruvate, lactate, glutamate and malate induced:**<br>energy sources and supply for ATP necessary for sperm motility	
female Sprague Dawley rats	α-KG	250 mg/kg/day	Intragastric administration	**Decreased/reduced expression in/of:** NLRP3, GSDMD, Caspase-1, IL-18, IL-1β<br>**Increased/improved in/of:** ovarian index, ovarian reserve, lactate, RLEs in the ovaries<br>**Induced:** serum hormone levels, follicle number	Liu *et al.* (2024)
C57BL/6 wild-type mESCs	dm-α-KG	4 mM	Addition to medium	**Induced:** maintenance of naïve pluripotency, PGC differentiation	Tischler *et al.* (2019)
male C57BL/6J mice<br>male OXGR1$^{Flox/Flox}$ mice<br>OXGR1-GKO mice	α-KG	2%<br>150 µM	Drinking water<br>Addition to medium	**Induced:** reversal of age- and heat-related epididymal sperm maturation disorders, intracellular Ca release from epididymal SMCs, pH_i reduction in the epididymal SMCs, increase of NBCe1 mRNA expression by α-KG/OXGR1 in the epididymal SMCs	Xu *et al.* (2022)
female C57BL/6 mice	dm-α-KG	3%	Containing food	**Induced:** maternal decidualization-based accumulation of α-KG due to activation of glutaminolysis, Gln-Glu-αKG enhancement flux support ATP generation during decidualization and decrease histone methylation, Glu-free diet decrease α-KG and impairs decidualization which increase the rate of fetal loss	Tang *et al.* (2023)
female ICR mice	dm-α-KG	1 mmol/L	Addition to medium	**Increased/improved in/of:** blastocyst formation in the presence of 0.01 mmol/L pyruvate (12%) with addition of dm-α-KG (49%), embryos-to-blastocysts (80%) development in the presence of 0.2 mmol/L pyruvate, with or without dm-α-KG, notable birth rate improvement in 0.2 mmol/L pyruvate and dm-α-KG (31.0%) compared with pyruvate alone (16.3%)<br>**Decreased/reduced in/of:** development beyond the two-cell stage of zygotes in medium lacking pyruvate, with or without dm-α-KG	Choi *et al.* (2019)
male and female C57BL/6J mice	dm-α-KG	0, 0.08, 0.4, 2, 10, 50 mM	Addition to medium	**Increased/improved in/of:** trophoblast invasion, embryo growth by IGF-1, GDF-15 secretion<br>**Induced:** downregulation of EGLN1-HIF-1α, early embryo implantation failure, SAB and/or FGR due to glutaminolysis blockage with GLS inhibitor BPTES or Glu dehydrogenase inhibitor EGCG, alleviation of pregnancy loss	Yang *et al.* (2022)
SCID mice/mESCs	dm-α-KG	0, 0.5, 1 mM	Addition to medium	**Induced:** self-renewal and pluripotency maintenance of mESCs based on the Psat1 regulation by OSN of α-KG amounts	Hwang *et al.* (2016)

C57BL/6 JAX 000664/ 129S4/SvJae JAX 009104 mice/mESCs	dm-α-KG	0.5, 1, 2, 3, 4 mM	Addition to medium	**Induced:** α-KG/succinate ratio maintains self-renewal and pluripotency of naïve mESCs	Carey *et al.* (2015)
naïve v6.5 mESCs/mEpiSCs	α-KG/dm-α-KG	0, 4, 8, 12 mM	Addition to medium	**Induced:** increased α-KG or α-KG/succinate ratio accelerates, while a high succinate level impedes primed PSCs differentiation, which indicates the pivotal role of α-KG for self-renewal and differentiation that rely on the pluripotent state	Xing *et al.* (2020)
mESCs line bearing Venus-tagged *Blimp1*	dm-α-KG	4 mmol/L	Addition to medium	**Induced:** α-KG reverses glutaminolysis inhibition by rescuing PGCLC due to BPTES	TeSlaa *et al.* (2016)

dm – dimethyl; ATP – adenosine triphosphate; CG – cortical granule; GSH – glutathione; ROS – reactive oxygen species; DNA – deoxyribonucleic acid; OCT4 – octamer-binding transcription factor 4; NANOG – homebox protein NANOG; SOX2 – sex determining region Y-box 2; Bcl2 – B-cell lymphoma 2; Nrf – nuclear factor erythroid 2-related factor 2; ARE – antioxidant response element; $AlCl_3$ – aluminum; Fe – iron; TAG – triacylglyceride; OS – oxidative stress; H_2O_2 – hydrogen peroxide; HSP90 – heat shock protein 90; w^{Dah} – wild-type Dahomey; ADP – adenosine diphosphate; HDAC4 – histone deacetylase 4; PI3K – phosphoinositide 3-kinase; TORC – target of rapamycin complex; PGC – progastricsin; SREBP – sterol regulatory element-binding protein; Hsp22 – heat shock protein 22; Hsp70 – heat shock protein 70; cry – cryptochrome; FoxO – forkhead box protein O; HNF4 - hepatocyte nuclear factor 4; p300 - histone acetyltransferase p300; Sirt1 - silent mating type information regulation 2 homolog 1; AMPKα – AMP-activated protein kinase subunit alpha; mTOR – mammalian target of rapamycin; BMI – body mass index; RLE – rate-limiting enzyme; FSH - follicle-stimulating hormone; $O_2^{\cdot -}$ – superoxide anion; NLRP3 – NOD-like receptor family pyrin domain-containing 3; GSDMD – gasdermin D IL-18 – interleukin-18; IL-1β – interleukin-1β; mESC – mouse embryonic stem cell; PGC – primordial germ cell; Ca – calcium; SMC – smooth muscle cell; NBCe1 – Na+/HCO3 - cotransporter; mRNA – messenger ribonucleic acid; OXGR1 – 2-oxoglutarate receptor 1; Gln – glutamine; Glu – glutamate; IGF-1 – insulin-like growth factor-1; GDF-15 – growth differentiation factor-15; EGLN1 – Egl nine homolog 1; HIF-1α – hypoxia inducible factor-1α; SAB – spontaneous abortion; FGR – fetal growth restriction; GLS – glutaminase; BPTES – Bis-2-(5-phenylacetamido-1,3,4-thiadiazol-2-yl)ethyl sulfide; EGCG – epigallocatechin gallate sulfate; SCID - severe combined immunodeficiency; Psat1 – phosphoserine aminotransferase 1; OSN – Oct4/Sox2/Nanog; PSC – primordial stem cell; mEpiSC – mouse epiblast stem cell; PGCLC – primordial germ cell-like cell

REFERENCES

Bayliak MM, Lylyk MP, Shmihel HV, Sorochynska OM, Semchyshyn OI, Storey JM, Storey KB & Lushchak VI 2017 Dietary alpha-ketoglutarate promotes higher protein and lower triacylglyceride levels and induces oxidative stress in larvae and young adults but not in middle-aged Drosophila melanogaster. *Comparative Biochemistry and Physiology. Part A, Molecular and Integrative Physiology* **204** 28–39. (https://doi.org/10.1016/j.cbpa.2016.11.005)

Bayliak MM, Lylyk MP, Gospodaryov DV, Kotsyubynsky VO, Butenko NV, Storey KB & Lushchak VI 2019 Protective effects of alpha-ketoglutarate against aluminum toxicity in Drosophila melanogaster. Comparative Biochemistry and Physiology. *Toxicology and Pharmacology* **217** 41–53. (https://doi.org/10.1016/j.cbpc.2018.11.020)

Breininger E, Vecchi Galenda BE, Alvarez GM, Gutnisky C & Cetica PD 2014 Phosphofructokinase and malate dehydrogenase participate in the in vitro maturation of porcine oocytes. *Reproduction in Domestic Animals* **49** 1068–1073. (https://doi.org/10.1111/rda.12437)

Carey BW, Finley LWS, Cross JR, Allis CD & Thompson CB 2015 Intracellular α-ketoglutarate maintains the pluripotency of embryonic stem cells. *Nature* **518** 413–416. (https://doi.org/10.1038/nature13981)

Chen Q, Gao L, Li J, Yuan Y, Wang R, Tian Y & Lei A 2022 α-Ketoglutarate improves meiotic maturation of porcine oocytes and promotes the development of PA embryos, potentially by reducing oxidative stress through the Nrf2 pathway. *Oxidative Medicine and Cellular Longevity* **2022** 7113793. (https://doi.org/10.1155/2022/7113793)

Choi ES, Kawano K, Hiraya M, Matsukawa E & Yamada M 2019 Effects of pyruvate and dimethyl-α-ketoglutarate, either alone or in combination, on pre- and post-implantation development of mouse zygotes cultured in vitro. *Reproductive Medicine and Biology* **18** 405–410. (https://doi.org/10.1002/rmb2.12288)

Hao Y, Wang J, Ren J, Liu Z, Bai Z, Liu G & Dai Y 2022 Effect of dimethyl alpha-ketoglutarate supplementation on the in vitro developmental competences of ovine oocytes. *Theriogenology* **184** 171–184. (https://doi.org/10.1016/j.theriogenology.2022.03.013)

Hwang I-Y, Kwak S, Lee S, Kim H, Lee SE, Kim J-H, Kim YA, Jeon YK, Chung DH, Jin X, et al. 2016 Psat1-dependent fluctuations in α-ketoglutarate affect the timing of ESC differentiation. *Cell Metabolism* **24** 494–501. (https://doi.org/10.1016/j.cmet.2016.06.014)

Li S-F, Liu H-X, Zhang Y-B, Yan Y-C & Li Y-P 2010 The protective effects of alpha-ketoacids against oxidative stress on rat spermatozoa in vitro. *Asian Journal of Andrology* **12** 247–256. (https://doi.org/10.1038/aja.2009.78)

Li T, Liu J, Liu K, Wang Q, Cao J, Xiao P, Yang W, Li X, Li J, Li M, et al. 2023 Alpha-ketoglutarate ameliorates induced premature ovarian insufficiency in rats by inhibiting apoptosis and upregulating glycolysis. *Reproductive Biomedicine Online* **46** 673–685. (https://doi.org/10.1016/j.rbmo.2023.01.005)

Liu K, Wu Y, Yang W, Li T, Wang Z, Xiao S, Peng Z, Li M, Xiong W, Li M, et al. 2024 α-Ketoglutarate Improves Ovarian Reserve Function in Primary Ovarian Insufficiency by Inhibiting NLRP3-Mediated pyroptosis of Granulosa Cells. *Molecular Nutrition and Food Research* **68** e2300784. (https://doi.org/10.1002/mnfr.202300784)

Lylyk MP, Bayliak MM, Shmihel HV, Storey JM, Storey KB & Lushchak VI 2018 Effects of alpha-ketoglutarate on lifespan and functional aging of Drosophila melanogaster flies. *Ukrainian Biochemical Journal* **90** 49–61. (https://doi.org/10.15407/ubj90.06.049)

Su Y, Wang T, Wu N, Li D, Fan X, Xu Z, Mishra SK & Yang M 2019 Alphaketoglutarate extends drosophila lifespan by inhibiting mTOR and activating AMPK. *Aging* **11** 4183–4197. (https://doi.org/10.18632/aging.102045)

Tang L, Xu X-H, Xu S, Liu Z, He Q, Li W, Sun J, Shuai W, Mao J, Zhao J-Y, et al. 2023 Dysregulated Gln-Glu-α-ketoglutarate axis impairs maternal decidualization and increases the risk of recurrent spontaneous miscarriage. *Cell Reports. Medicine* **4** 101026. (https://doi.org/10.1016/j.xcrm.2023.101026)

TeSlaa T, Chaikovsky AC, Lipchina I, Escobar SL, Hochedlinger K, Huang J, Graeber TG, Braas D & Teitell MA 2016 α-Ketoglutarate accelerates the initial differentiation of primed human pluripotent stem cells. *Cell Metabolism* **24** 485–493. (https://doi.org/10.1016/j.cmet.2016.07.002)

Tischler J, Gruhn WH, Reid J, Allgeyer E, Buettner F, Marr C, Theis F, Simons BD, Wernisch L & Surani MA 2019 Metabolic regulation of pluripotency and germ cell fate through α-ketoglutarate. *EMBO Journal* **38** e99518. (https://doi.org/10.15252/embj.201899518)

Wang H, Xu J, Li H, Chen W, Zeng X, Sun Y & Yang Q 2023 Alphaketoglutarate supplementation ameliorates ovarian reserve and oocyte quality decline with aging in mice. *Molecular and Cellular Endocrinology* **571** 111935. (https://doi.org/10.1016/j.mce.2023.111935)

Xing M, Wang N, Zeng H & Zhang J 2020 α-ketoglutarate promotes the specialization of primordial germ cell-like cells through regulating epigenetic reprogramming. *Journal of Biomedical Research* **35** 36–46. (https://doi.org/10.7555/JBR.34.20190160)

Xu C, Yuan Y, Zhang C, Zhou Y, Yang J, Yi H, Gyawali I, Lu J, Guo S, Ji Y, et al. 2022 Smooth muscle AKG/OXGR1 signaling regulates epididymal fluid acid–base balance and sperm maturation. *Life Metabolism* **1** 67–80. (https://doi.org/10.1093/lifemeta/loac012)

Yang S-L, Tan H-X, Lai Z-Z, Peng H-Y, Yang H-L, Fu Q, Wang H-Y, Li D-J & Li M-Q 2022 An active glutamine/α-ketoglutarate/HIF-1α axis prevents pregnancy loss by triggering decidual IGF1+GDF15+NK cell differentiation. *Cellular and Molecular Life Sciences* **79** 611. (https://doi.org/10.1007/s00018-022-04639-x)

Zhang Z, He C, Gao Y, Zhang L, Song Y, Zhu T, Zhu K, Lv D, Wang J, Tian X, et al. 2021 α-ketoglutarate delays age-related fertility decline in mammals. *Aging Cell* **20** e13291. (https://doi.org/10.1111/acel.13291)
