# Supplementary material for: A scoping review regarding reproductive capacity modulation based on alpha-ketoglutarate supplementation
Source: Reproduction. 2024 Oct 7;168(5):e240137. doi: 10.1530/REP-24-0137 (PMC11558802; doi:10.1530/REP-24-0137)
Supplement: Supplementary File 1. Extensive searching strategies [file supplementary_file_1.pdf]

Supplementary File 1. Extensive searching strategies

<u>Primary keywords</u>

α-ketoglutaric acid, alpha-ketoglutaric acid, 2-ketoglutaric acid, 2-oxoglutaric acid, oxoglutaric acid, 2-oxopentanedioic acid, AKG, 2-oxoglutamate

1. PubMed-MEDLINE – United States (NLM, 1996)

<u>1.1. Detailed searching strings:</u>

((((((((((((α-ketoglutaric acid) OR (alpha-ketoglutaric acid)) OR (2-ketoglutaric acid)) OR (2-oxoglutaric acid)) OR (oxoglutaric acid)) OR (2-oxopentanedioic acid)) OR (AKG)) OR (2-oxoglutamate)) AND (reproductive system)) OR (fertility)) OR (reproduction)) AND (laboratory model)) OR (experimental animal)) NOT (humans) Filters: Clinical Trial, Veterinary, Observational Study, Veterinary, Other Animals, English, Female, MEDLINE, from 2010/1/1 - 2024/3/1

(((((("ketoglutaric acids"[MeSH Terms] OR ("ketoglutaric"[All Fields] AND "acids"[All Fields]) OR "ketoglutaric acids"[All Fields] OR ("alpha"[All Fields] AND "ketoglutaric"[All Fields] AND "acid"[All Fields]) OR "alpha ketoglutaric acid"[All Fields] OR ("ketoglutaric acids"[MeSH Terms] OR ("ketoglutaric"[All Fields] AND "acids"[All Fields]) OR "ketoglutaric acids"[All Fields] OR ("alpha"[All Fields] AND "ketoglutaric"[All Fields] AND "acid"[All Fields]) OR "alpha ketoglutaric acid"[All Fields]) OR ("ketoglutaric acids"[MeSH Terms] OR ("ketoglutaric"[All Fields] AND "acids"[All Fields]) OR "ketoglutaric acids"[All Fields] OR "2 ketoglutaric acid"[All Fields]) OR ("ketoglutaric acids"[MeSH Terms] OR ("ketoglutaric"[All Fields] AND "acids"[All Fields]) OR "ketoglutaric acids"[All Fields] OR "2 oxoglutaric acid"[All Fields]) OR ("oxoglutaric"[All Fields] AND ("acids"[MeSH Terms] OR "acids"[All Fields] OR "acid"[All Fields])) OR ("ketoglutaric acids"[MeSH Terms] OR ("ketoglutaric"[All Fields] AND "acids"[All Fields]) OR "ketoglutaric acids"[All Fields]) OR "AKG"[All Fields] OR "2-oxoglutamate"[All Fields]) AND ("genitalia"[MeSH Terms] OR "genitalia"[All Fields] OR ("reproductive"[All Fields] AND "system"[All Fields]) OR "reproductive system"[All Fields])) OR ("fertiles"[All Fields] OR "fertility"[MeSH Terms] OR "fertility"[All Fields] OR "fertile"[All Fields] OR "fertilities"[All Fields]) OR ("reproduction"[MeSH Terms] OR "reproduction"[All Fields] OR "reproductions"[All Fields] OR "reproductive"[All Fields] OR

"reproductively"[All Fields] OR "reproductives"[All Fields] OR "reproductivity"[All Fields])) AND (("laboratorial"[All Fields] OR "laboratories"[MeSH Terms] OR "laboratories"[All Fields] OR "laboratory"[All Fields] OR "laboratory s"[All Fields]) AND ("model"[All Fields] OR "model s"[All Fields] OR "modeled"[All Fields] OR "modeler"[All Fields] OR "modeler s"[All Fields] OR "modelers"[All Fields] OR "modeling"[All Fields] OR "modelings"[All Fields] OR "modelization"[All Fields] OR "modelizations"[All Fields] OR "modelize"[All Fields] OR "modelized"[All Fields] OR "modelled"[All Fields] OR "modeller"[All Fields] OR "modellers"[All Fields] OR "modelling"[All Fields] OR "modellings"[All Fields] OR "models"[All Fields]))) OR ("animals, laboratory"[MeSH Terms] OR ("animals"[All Fields] AND "laboratory"[All Fields]) OR "laboratory animals"[All Fields] OR ("experimental"[All Fields] AND "animal"[All Fields]) OR "experimental animal"[All Fields])) NOT ("human s"[All Fields] OR "humans"[MeSH Terms] OR "humans"[All Fields] OR "human"[All Fields])) AND ((medline[Filter]) AND (veterinaryclinicaltrial[Filter] OR veterinaryobservationalstudy[Filter]) AND (animal[Filter]) AND (female[Filter]) AND (2010/1/1:2024/3/1[pdat]) AND (english[Filter]))

<u>1.2. Filters applied for fields:</u>

ARTICLE TYPE: Clinical Trial, Veterinary, Observational Study, Veterinary

SPECIES: Other Animals

ARTICLE LANGUAGE: English

SEX: Female

OTHER: MEDLINE

2. Web of Science^{TM} (WOS) (Clarivate Analytics, 1997)

<u>2.1. Detailed searching strings:</u>

((((((((((((ALL=(α-ketoglutaric acid)) OR ALL=(alpha-ketoglutaric acid)) OR ALL=(2-ketoglutaric acid)) OR ALL=(2-oxoglutaric acid)) OR ALL=(oxoglutaric acid)) OR ALL=(2-oxopentanedioic acid)) OR ALL=(AKG)) OR ALL=(2-oxoglutamate)) AND ALL=(reproductive system)) OR ALL=(fertility)) OR ALL=(reproduction)) AND ALL=(laboratory model)) OR ALL=(experimental animal)) NOT ALL=(humans)

2.2. Filters applied for fields:

DOCUMENT TYPES: Article

WEB OF SCIENCE INDEX: Science Citation Index Expanded (SCI-Expanded)

LANGUAGE: English

WEB OF SCIENCE CATEGORIES: Developmental Biology

RESEARCH AREAS: Reproductive Biology

3. Scopus (Elsevier, 2004)

3.1. Detailed searching strings:

TITLE-ABS-KEY (α-ketoglutaric AND acid OR alpha-ketoglutaric AND acid OR 2-ketoglutaric AND acid OR 2-oxoglutaric AND acid OR oxoglutaric AND acid OR 2-oxopentanedioic AND acid OR akg OR 2-oxoglutamate) AND TITLE-ABS-KEY (reproductive AND system OR fertility OR reproduction) AND (LIMIT-TO (LANGUAGE , "English")) AND (LIMIT-TO (SRCTYPE , "j")) AND (LIMIT-TO (PUBSTAGE , "final")) AND (LIMIT-TO (DOCTYPE , "ar")) AND (LIMIT-TO (SUBJAREA , "MEDI") OR LIMIT-TO (SUBJAREA , "BIOC"))

SUBJECT AREA: Health Sciences – Veterinary (VETE): Biochemistry, Genetics and Molecular Biology, Medicine

3.2. Filters applied for fields:

DOCUMENT TYPE: Article

PUBLICATION STAGE: Final

SOURCE TYPE: Journal

LANGUAGE: English

4. Excerpta Medica dataBASE (EMBASE) (Elsevier, 1947)

4.1. Detailed searching strings:

((((((('α ketoglutaric' AND ('acid' OR 'acid'/exp OR acid) OR 'alpha ketoglutaric') AND ('acid' OR 'acid'/exp OR acid) OR '2 ketoglutaric') AND ('acid' OR 'acid'/exp OR acid) OR '2 oxoglutaric') AND ('acid' OR 'acid'/exp OR acid) OR oxoglutaric) AND ('acid' OR 'acid'/exp OR acid) OR '2 oxopentanedioic') AND ('acid' OR 'acid'/exp OR acid) OR akg OR '2 oxoglutamate') AND [article]/lim AND [english]/lim AND [female]/lim AND ([animal cell]/lim OR [animal

experiment]/lim OR [animal model]/lim OR [animal tissue]/lim) AND [animals]/lim AND [embase]/lim

4.2. Filters applied for fields:

ANIMAL STUDY TYPES: Animal Cell, Animal Experiment, Animal Model, Animal Tissue

GENDER: Female

LANGUAGES: English

PUBLICATION TYPES: Article

QUICK LIMITS: Animals

SOURCES: Embase

MAPPING: Map to preferred term in Emtree, Search also as free text in all fields, Explode using narrower Emtree terms, Search as broadly as possible
