# Supplementary material for: A scoping review regarding reproductive capacity modulation based on alpha-ketoglutarate supplementation
Source: Reproduction. 2024 Oct 7;168(5):e240137. doi: 10.1530/REP-24-0137 (PMC11558802; doi:10.1530/REP-24-0137)
Supplement: Supplementary File 2. Cumulative list of entries based on the year of publication and database [file supplementary_file_2.pdf]

Supplementary File 2. Cumulative list of entries based on the year of publication and database

	Databases			
**Year of publication**	**PubMed**	**WOS**	**Scopus**	**EMBASE**
2010	-	19	-	7
2011	-	26	-	3
2012	-	18	-	11
2013	-	23	-	7
2014	-	17	-	9
2015	-	15	-	10
2016	-	37	-	17
2017	5	36	-	23
2018	81	22	-	16
2019	176	33	-	31
2020	167	36	-	35
2021	157	31	1	42
2022	155	17	1	35
2023	179	22	1	40
2024	27	2	1	15
**Cumulative**	**947**	**354**	**4**	**301**
**Total**	**1,606**			
