# Supplementary material for: A scoping review regarding reproductive capacity modulation based on alpha-ketoglutarate supplementation
Source: Reproduction. 2024 Oct 7;168(5):e240137. doi: 10.1530/REP-24-0137 (PMC11558802; doi:10.1530/REP-24-0137)
Supplement: Supplementary File 3. Eligibility assessment [file supplementary_file_3.pdf]

Supplementary File 3. Eligibility assessment

List of manuscripts examined during the first step of assessment

Article title	Eligible or not eligible?	Reason
[Energy Metabolism in the Placenta and the Role of Disturbances in the Development of Placental Insufficiency at an Exacerbation of Cytomegalovirus Infection]	**Not eligible**	**Other language - Russian**
Dimethyl α-ketoglutarate inhibits maladaptive autophagy in pressure overload-induced cardiomyopathy	**Not eligible**	**Out of scope**
Triggering and amplification of insulin secretion by dimethyl alpha-ketoglutarate, a membrane permeable alpha-ketoglutarate analogue	**Not eligible**	**Out of scope**
Testicular ACE regulates sperm metabolism and fertilization through the transcription factor PPARγ	**Not eligible**	**No direct correlation**
A proteomics-metabolomics approach indicates changes in hypothalamic glutamate-GABA metabolism of adult female rats submitted to intrauterine growth restriction	**Not eligible**	**No direct correlation**
The Effects of Prenatal Supplementation with β-Hydroxy-β-Methylbutyrate and/or Alpha-Ketoglutaric Acid on the Development and Maturation of Mink Intestines Are Dependent on the Number of Pregnancies and the Sex of the Offspring	**Not eligible**	**No direct correlation**
AKT signaling is associated with epigenetic reprogramming via the upregulation of TET and its cofactor, alpha-ketoglutarate during iPSC generation	**Not eligible**	**No direct correlation**
Abnormal early folliculogenesis due to impeded pyruvate metabolism in mouse oocytes†	**Not eligible**	**No direct correlation**
Alpha-ketoglutarate supplementation ameliorates ovarian reserve and oocyte quality decline with aging in mice	**Eligible**	**Introduced in the manuscript**
Smooth muscle AKG/OXGR1 signaling regulates epididymal fluid acid–base balance and sperm maturation	**Eligible**	**Introduced in the manuscript**
α-ketoglutarate delays age-related fertility decline in mammals	**Eligible**	**Introduced in the manuscript**
α-Ketoglutarate Improves Ovarian Reserve Function in Primary Ovarian Insufficiency by Inhibiting NLRP3-Mediated Pyroptosis of Granulosa Cells	**Eligible**	**Introduced in the manuscript**
Effects of pyruvate and dimethyl-α-ketoglutarate, either alone or in combination, on pre- and post-implantation development of mouse zygotes cultured in vitro	**Eligible**	**Introduced in the manuscript**
α-Ketoglutarate Accelerates the Initial Differentiation of Primed Human Pluripotent Stem Cells	**Eligible**	**Introduced in the manuscript**
Effect of dimethyl alpha-ketoglutarate supplementation on the in vitro developmental competences of ovine oocytes	**Eligible**	**Introduced in the manuscript**
α-ketoglutarate promotes the specialization of primordial germ cell-like cells through regulating	**Eligible**	**Introduced in the**

epigenetic reprogramming		**manuscript**
Maternal high-fat diet changes DNA methylation in the early embryo by disrupting the TCA cycle intermediary alpha ketoglutarate	**Eligible**	**Introduced in the manuscript**
Alpha-ketoglutarate extends Drosophila lifespan by inhibiting mTOR and activating AMPK	**Eligible**	**Introduced in the manuscript**
α-Ketoglutarate Improves Meiotic Maturation of Porcine Oocytes and Promotes the Development of PA Embryos, Potentially by Reducing Oxidative Stress through the Nrf2 Pathway	**Eligible**	**Introduced in the manuscript**
Alpha-ketoglutarate affects murine embryo development through metabolic and epigenetic modulations	**Eligible**	**Introduced in the manuscript**
Alpha-ketoglutarate ameliorates induced premature ovarian insufficiency in rats by inhibiting apoptosis and upregulating glycolysis	**Eligible**	**Introduced in the manuscript**
An active glutamine/α-ketoglutarate/HIF-1α axis prevents pregnancy loss by triggering decidual IGF1+GDF15+NK cell differentiation	**Eligible**	**Introduced in the manuscript**
Dysregulated Gln-Glu-α-ketoglutarate axis impairs maternal decidualization and increases the risk of recurrent spontaneous miscarriage	**Eligible**	**Introduced in the manuscript**
The protective effects of alpha-ketoacids against oxidative stress on rat spermatozoa in vitro	**Eligible**	**Introduced in the manuscript**
Metabolic regulation of pluripotency and germ cell fate through α-ketoglutarate	**Eligible**	**Introduced in the manuscript**
Dietary alpha-ketoglutarate promotes higher protein and lower triacylglyceride levels and induces oxidative stress in larvae and young adults but not in middle-aged Drosophila melanogaster	**Eligible**	**Introduced in the manuscript**
Intracellular α-ketoglutarate maintains the pluripotency of embryonic stem cells	**Eligible**	**Introduced in the manuscript**
Psat1-Dependent Fluctuations in α-Ketoglutarate Affect the Timing of ESC Differentiation	**Eligible**	**Introduced in the manuscript**
Protective effects of alpha-ketoglutarate against aluminum toxicity in Drosophila melanogaster	**Eligible**	**Introduced in the manuscript**
Effects of alpha-ketoglutarate on lifespan and functional aging of Drosophila melanogaster flies	**Eligible**	**Introduced in the manuscript**
Phosphofructokinase and malate dehydrogenase participate in the in vitro maturation of porcine oocytes	**Eligible**	**Introduced in the manuscript**

List of manuscripts examined during the second step of assessment

Maternal high-fat diet changes DNA methylation in the early embryo by disrupting the TCA	**Not eligible**	**Could not be accessed**

cycle intermediary alpha ketoglutarate		
Alpha-ketoglutarate affects murine embryo development through metabolic and epigenetic modulations	**Not eligible**	**Could not be accessed**
Effect of dimethyl alpha-ketoglutarate supplementation on the in vitro developmental competences of ovine oocytes	**Eligible**	**Introduced in the manuscript**
α-Ketoglutarate Improves Meiotic Maturation of Porcine Oocytes and Promotes the Development of PA Embryos, Potentially by Reducing Oxidative Stress through the Nrf2 Pathway	**Eligible**	**Introduced in the manuscript**
Phosphofructokinase and malate dehydrogenase participate in the in vitro maturation of porcine oocytes	**Eligible**	**Introduced in the manuscript**
Protective effects of alpha-ketoglutarate against aluminum toxicity in Drosophila melanogaster	**Eligible**	**Introduced in the manuscript**
Dietary alpha-ketoglutarate promotes higher protein and lower triacylglyceride levels and induces oxidative stress in larvae and young adults but not in middle-aged Drosophila melanogaster	**Eligible**	**Introduced in the manuscript**
Effects of alpha-ketoglutarate on lifespan and functional aging of Drosophila melanogaster flies	**Eligible**	**Introduced in the manuscript**
Alpha-ketoglutarate extends Drosophila lifespan by inhibiting mTOR and activating AMPK	**Eligible**	**Introduced in the manuscript**
Alpha-ketoglutarate supplementation ameliorates ovarian reserve and oocyte quality decline with aging in mice	**Eligible**	**Introduced in the manuscript**
α-ketoglutarate delays age-related fertility decline in mammals	**Eligible**	**Introduced in the manuscript**
Alpha-ketoglutarate ameliorates induced premature ovarian insufficiency in rats by inhibiting apoptosis and upregulating glycolysis	**Eligible**	**Introduced in the manuscript**
The protective effects of alpha-ketoacids against oxidative stress on rat spermatozoa in vitro	**Eligible**	**Introduced in the manuscript**
α-Ketoglutarate Improves Ovarian Reserve Function in Primary Ovarian Insufficiency by Inhibiting NLRP3-Mediated Pyroptosis of Granulosa Cells	**Eligible**	**Introduced in the manuscript**
Metabolic regulation of pluripotency and germ cell fate through α-ketoglutarate	**Eligible**	**Introduced in the manuscript**
Smooth muscle AKG/OXGR1 signaling regulates epididymal fluid acid–base balance and sperm maturation	**Eligible**	**Introduced in the manuscript**
Dysregulated Gln-Glu-α-ketoglutarate axis impairs maternal decidualization and increases the risk of recurrent spontaneous miscarriage	**Eligible**	**Introduced in the manuscript**
Effects of pyruvate and dimethyl-α-ketoglutarate, either alone or in combination, on pre- and post-implantation development of mouse zygotes cultured in vitro	**Eligible**	**Introduced in the manuscript**

An active glutamine/α-ketoglutarate/HIF-1α axis prevents pregnancy loss by triggering decidual IGF1+GDF15+NK cell differentiation	**Eligible**	**Introduced in the manuscript**
Psat1-Dependent Fluctuations in α-Ketoglutarate Affect the Timing of ESC Differentiation	**Eligible**	**Introduced in the manuscript**
Intracellular α-ketoglutarate maintains the pluripotency of embryonic stem cells	**Eligible**	**Introduced in the manuscript**
α-ketoglutarate promotes the specialization of primordial germ cell-like cells through regulating epigenetic reprogramming	**Eligible**	**Introduced in the manuscript**
α-Ketoglutarate Accelerates the Initial Differentiation of Primed Human Pluripotent Stem Cells	**Eligible**	**Introduced in the manuscript**

Final list of manuscripts included

Effect of dimethyl alpha-ketoglutarate supplementation on the in vitro developmental competences of ovine oocytes	**Eligible**	**Introduced in the manuscript**
α-Ketoglutarate Improves Meiotic Maturation of Porcine Oocytes and Promotes the Development of PA Embryos, Potentially by Reducing Oxidative Stress through the Nrf2 Pathway	**Eligible**	**Introduced in the manuscript**
Phosphofructokinase and malate dehydrogenase participate in the in vitro maturation of porcine oocytes	**Eligible**	**Introduced in the manuscript**
Protective effects of alpha-ketoglutarate against aluminum toxicity in Drosophila melanogaster	**Eligible**	**Introduced in the manuscript**
Dietary alpha-ketoglutarate promotes higher protein and lower triacylglyceride levels and induces oxidative stress in larvae and young adults but not in middle-aged Drosophila melanogaster	**Eligible**	**Introduced in the manuscript**
Effects of alpha-ketoglutarate on lifespan and functional aging of Drosophila melanogaster flies	**Eligible**	**Introduced in the manuscript**
Alpha-ketoglutarate extends Drosophila lifespan by inhibiting mTOR and activating AMPK	**Eligible**	**Introduced in the manuscript**
Alpha-ketoglutarate supplementation ameliorates ovarian reserve and oocyte quality decline with aging in mice	**Eligible**	**Introduced in the manuscript**
α-ketoglutarate delays age-related fertility decline in mammals	**Eligible**	**Introduced in the manuscript**
Alpha-ketoglutarate ameliorates induced premature ovarian insufficiency in rats by inhibiting apoptosis and upregulating glycolysis	**Eligible**	**Introduced in the manuscript**
The protective effects of alpha-ketoacids against oxidative stress on rat spermatozoa in vitro	**Eligible**	**Introduced in the manuscript**

α-Ketoglutarate Improves Ovarian Reserve Function in Primary Ovarian Insufficiency by Inhibiting NLRP3-Mediated Pyroptosis of Granulosa Cells	**Eligible**	**Introduced in the manuscript**
Metabolic regulation of pluripotency and germ cell fate through α-ketoglutarate	**Eligible**	**Introduced in the manuscript**
Smooth muscle AKG/OXGR1 signaling regulates epididymal fluid acid–base balance and sperm maturation	**Eligible**	**Introduced in the manuscript**
Dysregulated Gln-Glu-α-ketoglutarate axis impairs maternal decidualization and increases the risk of recurrent spontaneous miscarriage	**Eligible**	**Introduced in the manuscript**
Effects of pyruvate and dimethyl-α-ketoglutarate, either alone or in combination, on pre- and post-implantation development of mouse zygotes cultured in vitro	**Eligible**	**Introduced in the manuscript**
An active glutamine/α-ketoglutarate/HIF-1α axis prevents pregnancy loss by triggering decidual IGF1+GDF15+NK cell differentiation	**Eligible**	**Introduced in the manuscript**
Psat1-Dependent Fluctuations in α-Ketoglutarate Affect the Timing of ESC Differentiation	**Eligible**	**Introduced in the manuscript**
Intracellular α-ketoglutarate maintains the pluripotency of embryonic stem cells	**Eligible**	**Introduced in the manuscript**
α-ketoglutarate promotes the specialization of primordial germ cell-like cells through regulating epigenetic reprogramming	**Eligible**	**Introduced in the manuscript**
α-Ketoglutarate Accelerates the Initial Differentiation of Primed Human Pluripotent Stem Cells	**Eligible**	**Introduced in the manuscript**
